# Small Steps, Big Vision: using multi-stage qualitative research to develop a grab-and-go guide to support utilisation of the Ambitions for Palliative and End of Life Care framework

**DOI:** 10.1186/s12904-024-01466-8

**Published:** 2024-06-14

**Authors:** Erica Borgstrom, Joanne Jordan, Una St Ledger, Claire Henry

**Affiliations:** Faculty of Wellbeing, Education and Language Studies, The Open University, Walton Hall, Milton Keynes, MK7 6AA UK

**Keywords:** End of life care, Hospice care, Education and training, Terminal care, Policy, Policy implementation, Knowledge exchange

## Abstract

**Background:**

The Ambitions for Palliative and End of Life Care is a national framework for local action in England co-produced by over 30 partners; little research has been conducted on how the Framework is received and used. This study sought to examine and support how people understand, interpret, and implement the Framework.

**Methods:**

A multi-stage qualitative methodology involving four stages of data collection: (1) case study interviews, (2) focus groups, (3) interactive workshops, and (4) Evidence Cafés. From initial interviews, ongoing thematic data analysis informed the design and focus of subsequent stages as part of a process of knowledge transfer.

**Results:**

A practical resource to support service provision and development was produced; a grab-and-go guide called “Small Steps, Big Visions”. It focuses on the eight foundations in the Ambitions Framework, with additional guidance on collaboration and partnership working, and sharing learning. Each foundation is presented with a ‘what’ (definition), ‘ask’ (prompt questions), and ‘examples in action’ (drawn from case studies).

**Conclusions:**

Research can contribute to policy implementation to advance palliative and end of life care. The engagement and input of those responsible for implementation is key.

**Supplementary Information:**

The online version contains supplementary material available at 10.1186/s12904-024-01466-8.

## Introduction

Originally released in 2015, the relaunched *Ambitions for Palliative and End of Life Care: a national framework for local action (2021–2026)* (“Framework”) [1] provides a vision for palliative and end of life care (PEOLC) at the local level throughout England. Developed in collaboration with over thirty cross-sector partners, the Framework sets out six Ambitions, accompanied by a set of Foundations that identify what is needed for their realisation (Table 1). Although not mandated policy, the Framework is incorporated into the PEOLC Guidance for Integrated Care Boards (ICBs), reflecting their statutory duties under the Health and Care Act (2022) [2]. The Framework is accompanied by a self-assessment tool [3], which can be used to self-assess provision against the Ambitions.

**Table 1:** Six Ambitions and eight Foundations

Ambition	Foundations of all Ambitions
Ambition One: Each person is seen as an individual	1. Personalised care planning 2. Shared records 3. Education and training 4. 24/7 access 5. Evidence and information 6. Involving, supporting and caring for those important to the dying person 7. Co-design 8. Leadership
Ambition Two: Each person gets fair access to care	
Ambition Three: Maximising comfort and wellbeing	
Ambition Four: Care is coordinated	
Ambition Five: All staff are prepared to care	
Ambition Six: Each community is prepared to help	

Editorials [4, 5] and blogs [6–8] have endorsed the Framework and examples of its use are documented [9–11]. However, research evidence is limited. Barker’s work [12, 13] with professionals involved in PEOLC policy development demonstrated appreciation of the Framework in pulling together various policy strands and providing a direction for local-level service development. However, its understandability was questioned, and concerns raised regarding the Framework’s limitations as non-mandatory guidance, as well as potential to increase inequalities nationally. Our recent survey of PEOLC providers [14, 15] found a similar pattern of understanding. Although the Framework was endorsed as a validation of the importance of PEOLC and statement of excellence in provision, uncertainty over how this excellence might be achieved was evident. In addition, concerns were raised that differential uptake of the Ambitions would exacerbate local-level inequalities in provision. Our survey [14, 15] also demonstrated a clear desire from practitioners for support to help address identified challenges, particularly concerning implementation of the Ambitions.

The difficulty with implementing policy and guidance in health care is well documented [16, 17]. Firstly, implementation is a complex process, especially when a wide range of stakeholders operating across different settings are involved [18]. Secondly, the large number of policies and guidance that practitioners need to embed can create challenges. In the late 1990s, it was noted that clinicians were ‘inundated by a tidal wave of guidelines’ [19], and the volume of PEOLC policies and guidance has grown nationally and internationally since the early 2000s [20, 21]. While some of the health services research literature focuses on the development and implementation of evidence-based guidelines (see for example [22, 23]), our interest is in documents – like the Framework – that are viewed as guidance for service development and practice. Such documents can have an ambiguous role in healthcare as they are not formal policy, nor are they protocols for patient care (unlike evidence-based guidelines). Boundary objects – an artefact or practical resource that facilitates the sharing of ideas across social worlds, such as that involving policymakers and practitioners– have been identified as having the potential to transform knowledge and practice [24] including in PEOLC [25]. It is also recognised that successful implementation can be supported by a toolkit, which can include boundary objects that help explain what people are supposed to know, how they can do things differently, and to see how the change may fit with their organisational context [26]. There is, however, very little existing literature on how to develop such objects; the focus tends to be on guidelines or best practice (see for example [27]), or on the development of toolkits for specific clinical interventions [28].

To meet the desire for support with the implementation of the Ambitions Framework and building on our previous mapping survey research, we undertook a multi-phased study, which examined how people interpreted and used the Framework [29]. The study led to the development of a practical resource (a Grab and Go Guide, “Guide”), designed to help users to identify and undertake action in pursuit of the Ambitions.^1^ It was beyond the scope of the study to build a full toolkit for Framework implementation; additionally, there are some existing resources hosted on NHS England. Instead, we drew on the findings of our study to deliver a boundary object that provided additional support with implementation. This article describes the process through which this object (Guide) was developed and discusses the implications for research, policy and practice. As such it does not report detailed findings from our multi-phased study; these are reported elsewhere [29].

## Methods

**Fig. 1:**
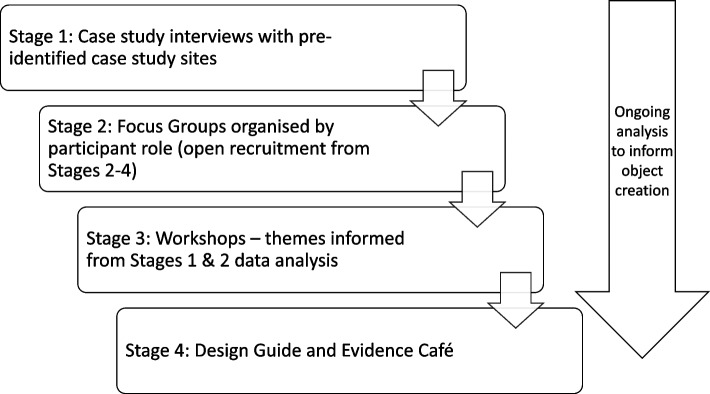
Study Design

This was a four, inter-dependent staged qualitative study (see Fig. 1). Developing toolkits is a multi-staged process involving multiple methods and stakeholder engagement [28]. Since we were aiming to only create one object (limited due to resource constraints), we adapted the toolkit development resource produced by the University of California Berkeley School of Social Welfare (CalSWEC) [26]. CalSWEC describe a 9 step process to develop an implementation toolkit: investigate, define, engage, assess, plan, transfer learning, evaluate, consider policy impact, and consider fiscal impact. Although designed for Human Services, others have also adapted the resource for healthcare contexts [28]. Our use of the CalSWEC process was driven by the data collected during our study and is described below. All data collection was online via MS Teams; with consent, sessions were recorded and auto-transcribed before being anonymised. The study ran from April 2022 to March 2023; data collection was undertaken between May 2022 and January 2023. Ethical approval was received from the Human Research Ethics Committee at The Open University (HREC/4304/Borgstrom).

The study team was not involved in the development or relaunch of the Framework. The team is interdisciplinary and is made up of two social scientists (EB, JJ) and two nurses (USL, CH). EB has been researching English PEOLC policy and guidance since 2010. JJ is experienced in evaluating PEOLC services and engaging a wide range of stakeholders for knowledge exchange. USL has specialist expertise in healthcare professional education as well as researching professional’s experiences of service provision. CH has over 30 years of experience in clinical, managerial and quality improvement within the NHS and third sector, as well as PEOLC policy-making and implementation on a national scale. Collectively, the team brought insights from their respective disciplines and experiences to inform the study design and the design of the object (Guide). Regular team meetings were held to discuss ongoing data collection and analysis, and were supported by an advisory group which included academics, PEOLC service providers, and members of the public (as representatives). This combination allowed us to foreground focusing on research and practice gaps in the study as they emerged within the wider recognition that no evaluation of the Framework had been done to date, and to question ‘what is useful in practice’ as a driver for both the design and focus of the study research stages, as well as the development of the final object (Guide).

Stage 1 involved 17 in-depth case study interviews with participants who had used the Framework in local service development. Participants were identified from the previous mapping survey [14, 15] and/or invited by the research team, purposively selected for diversity in terms of locality, setting and type of work undertaken. Interviews were selected for the case study component to enable participants dedicated, confidential time to discuss the Framework, their service/practice, and other issues they considered relevant to implementing policy/guidance or service development. Using a semi-structured guide, discussion focused on how the Framework had been used, issues involved in doing so, and the impact of activity undertaken. Where available, interviewees provided written material capturing their use of the Framework (e.g. education slide decks used in training, or local strategy documents). Data analysis followed a dual deductive and inductive process. Deductively, we used the core question “What do the data suggest for how the Framework is being implemented, and what are the key issues involved?” as the point of entry into the data. Inductively, we interrogated the data for what they were suggesting in relation to our guiding question, using a three-step process. This process involved: an initial categorisation of the data according to their essential content; comparing the different sections of the data created through step one to identify how they connected; and, expressing this connection through the development of overarching themes and sub-themes. Analysis was undertaken by two members of the team (EB, CH) who collaboratively reviewed all interview transcripts. Based on this process, we identified four major themes and attendant sub-themes: (a) major theme – how implemented (sub-themes: why the chosen focus of work; activity undertaken); (b) major theme – challenges and opportunities (sub-themes: how resolved or capitalised on; to what effect); (c) major theme – outcomes achieved (sub-themes: impact; how measured/identified); and (d) major theme – perceived implications for use of the Framework (sub-themes: requirements for implementation; how use of the Framework related to other areas of service provision).

Stage 2 involved four focus groups, organised by ‘role’ – service managers, service providers, commissioners, and members of the public. Participants (*n* = 2–8; 21 in total) were drawn from the broader constituency of those involved in PEOLC, irrespective of whether they had used the Framework or had been selected for a case study interview. Stage 1 had provided us with understanding and experience from those who had directly engaged with the Ambitions Framework. Now, we wanted to create an opportunity to open up the conversation to others whose knowledge and experience were also relevant. We used homogenous focus groups to bring together groups of people with shared ‘location’ in terms of service provision and development within an interactive forum in which they could discuss, confirm and challenge us as researchers and one another. We used the themes identified in Stage 1 in the development of a semi-structured focus group interview guide. The guide facilitated discussion of issues that Stage 1 suggested were relevant, enabling participants to consider these issues according to their respective roles, responsibilities and priorities. We were careful to encourage participants with little or no experience of Framework implementation to offer their perspectives and for all participants to be able to raise issues not directly prompted by our questions.

Invitations were circulated to previous participants (with a request to disseminate), on social media, and where possible in relevant PEOLC networks/newsletters. Discussions lasted no more than 90 min. Analytically, we used a deductive approach, interrogating the data in terms of how it aligned with the thematic framework generated in Stage 1, but strategically taking account of new issues or variations on issues already identified. Areas of consensus and divergence in the group conversation were analysed closely to understand why they mattered to participants; areas of divergence enabled us to identify different understandings of the Framework and implementation. Analysis was undertaken collaboratively by the four members of the research team through discussion. This analysis showed overlap with what we had found in Stage 1 in terms of overarching themes, broadening the content of these themes (new sub-themes were developed) and capturing new perspectives on the constituent issues. For example, the theme of “challenges and opportunities” was expanded to include the ‘language and order of the Ambitions’, both of which could be experienced as ambiguous, leading to uncertainty about what constituted relevant work and if and how it should be prioritised. The theme of “perceived implications for use of the Framework” was consolidated; in Stage 1 participants had talked about their use of the Foundations set out in the Framework in their implementation activity; Stage 2 participants also highlighted the value of using the Foundations as they provided a tangible statement of activity relevant to the Ambitions.

By the end of Stage 2 we had accumulated an extensive body of evidence. At its core, this evidence showed acknowledgement of the Framework to advance the quality of PEOLC, identified areas of work considered particularly important, and revealed opportunities and challenges in progressing relevant activity. Our aim for Stage 3 was to move to consideration of how these areas of work and attendant opportunities and challenges might be addressed. To that end, for Stage 3, we structured it the data collection around four areas of work that had been consistently prioritised and led to questions about further implementation by participants in Stages 1 and 2: 1) sharing learning, 2) strategy, self-assessment, and measuring progress, 3) partnership working, and 4) notions of community in the context of PEOLC. Each area became the focus of a themed workshop (each with between 10–18 participants; 40 individuals in total). Recruitment for Stage 3 followed the recruitment strategy adopted for Stage 2. Individuals could attend as many workshops as they wished; some attended more than one, but no one attended all four. After initial provision of information on workshop aims, structure and content, a semi-structured guide facilitated discussion on key issues relating to each area. These included: relative importance of the area in terms of enabling Framework implementation; how it might be progressed; and, opportunities and challenges in doing so. Where relevant, participants shared examples of practice. Following the workshops, the research team met to review the findings. To enable us to move towards the development of an object, analysis of Stage 3 data differed from Stage 1 and 2 to prioritise a focus on facilitating implementation through an object. To do so, we interrogated the evidence by asking three key questions of it: ‘what is working well for services with regard to implementation of the Framework’; ‘where are there still barriers to implementation’, and ‘what could support further implementation’. As we undertook the review, we explicitly considered the evidence from Stage 3 in relation to that generated in Stages 1 and 2, looking for overlap, dissonance and implications for how implementation could be further progressed.

The review highlighted four key issues. Firstly, broad consensus around the value of the Ambitions Framework as a statement of high quality PEOLC and aspiration to advance service provision according to the values it enshrined. Secondly, wide variation in knowledge of, and confidence in ability to undertake action to advance, the Framework. Even where participants were knowledgeable, many reported not knowing where to start or how to progress. In part, this was linked to a perception of the Ambitions as important in setting out core principles and values for service development, but lacking a clear steer on how these might be translated into action. Some had used the Framework’s self-assessment tool; although the tool was considered practical for identifying gaps in service provision, participants found it difficult to use, especially when mapping more than one service. Moreover, once gaps were identified, how to address these gaps presented entirely new challenges, such as relevant action to take and how this action might achieve desired goals, given issues like limited resources, variable stakeholder buy-in and system-level priorities.

Thirdly, that focusing on one or more of the Foundations (rather than the Ambitions themselves) helped foster a sense of relevant action, often working towards the realisation of multiple Ambitions. As indicators of relevant areas of work to be addressed, the Foundations could be used as ‘levers’ for action, for example, when developing business cases or in the commissioning process, in ways that the Ambitions, as aspirations, could not. This approach was considered even more productive given the Foundations’ relevance to wider health and social care goals. Action taken in respect of the Foundations could therefore directly address the Ambitions within the wider system of working.

Finally, the importance of, and appetite for, sharing of practice and mutual learning across Ambitions-related work. Participants repeatedly asserted the value of knowledge exchange, to gain insight into the sort of action that might be taken, and how attendant opportunities and challenges might be addressed. In this context, they considered participation in the focus groups and workshops undertaken as part of our data collection an important source of learning. There were repeated requests for opportunities to continue this learning, to enable the development of knowledge pursuant to implementation.

Using this evidence, it was apparent that an object to help bridge the gap between the Framework and action that might be taken in response would be of value. Object development formed Stage 4 of our work, outlined below.

### Stage 4: Developing the object (Grab and Go Guide)

#### Preliminary work

The team used the insights from Stages 1–3 to help shape the focus and content of the object in four essential respects. Firstly, guidance on activity relevant to the Foundations; these offered a tangible focus for activity. Secondly, guidance on collaboration and partnership working, and sharing learning; these had emerged as priorities in respect of all action taken to progress the Foundations. Thirdly, the need to be as explicit as possible, whilst avoiding overly prescriptive guidance; it was important that users could see the possibilities for action in the context of their local service setting and priorities. Finally, how best to pitch content; at this stage, we were aiming to create an object relevant to the broad constituency of people (professional and non-professional) involved in PEOLC.

Using the CalSWEC categorisation of tools, we identified that a ‘definition tool’ would be beneficial as that would enable us to focus on defining the Foundations, adding information about sharing learning and collaboration, and keep the object relatively short. Definitional tools seek to explicitly define key aspects needed for implementation, including describing meanings and providing examples of implementation actions [26]. Development of the object also took into consideration existing Ambition Framework resources to avoid duplication. The decision to develop a short document was informed by evidence gathered in Stages 1–3 about how participants felt exiting resources were lengthy. Additionally, we were cognisant of the pragmatic need for a concise document, recognising how it may be used in busy day-to-day practice [30].

Based on the requirements identified, we developed the document using a three-part structure for action – ‘what, ask, and example in action’ – for each Foundation and for both collaboration and partnership working and sharing learning. The structure reflected our theoretical approach to supporting users to understand, interpret, and implement, all of which had been prioritised by participants throughout data collection, and are recognised as key components for implementation toolkits [26]. ‘What’ aids understanding by describing relevant activity, thereby unpacking its potential for action. ‘Ask’ is a series of prompts, which encourage users to think about (or interpret) what this potentially means for them in terms of their service and action, considering relevant activity already taken, to be progressed, or initiated.

When populating the ‘what’ element, we based content on participant stated need for clarity and inclusivity. The same approach informed the content of the ‘ask’ element, which reflected participant descriptions of the sorts of issues they had confronted, and associated questions they had asked themselves. The ‘Examples in Action’ were drawn from case studies collected during Stages 1–3, provided to illustrate a range of possible action, rather than examples of ‘best practice’ (since case studies were not evaluated). Adopting the ‘what, ask, and examples in action’ approach, we sought to create a tool that enabled people to access knowledge (often tacit) about each Foundation within their local-level context. Provisionally, we called our object a ‘Grab and Go Guide’, reflecting its succinctness and action-orientation, and used the title ‘Small Steps, Big Vision’ to link into how people thought about implementation in practice.

A draft of the Guide was shared initially with the study’s advisory group for feedback on the format adopted, language used, and examples provided. Feedback helped to refine the definitions, prompt questions, and examples provided. Resulting changes focused particularly on two components of the guidance: ‘co-design’ and ‘those important to the dying person’. Overall, refinement resulted in more consistent coverage of each of the Foundations and associated action.

#### Evidence cafes

We used the refined Guide during two Evidence Cafes [31], each with 6–15 participants and lasting two hours. An Evidence Café is a structured workshop designed to facilitate the process of translating research into practice by collecting different views on evidence and suggested outputs. They provide participants with an opportunity to explore how research can inform their practice, whilst also providing researchers with practice-based insights. Evidence Cafes are structured around one or more discussion objects rather than presentations to encourage knowledge exchange– in this study, the object was the Grab and Go Guide.

The aim of the Cafes was two-fold: (a) to garner comprehensive feedback on the draft Guide, on the basis of which final amendments could be made and (b) collect additional data on issues relating to use of the Guide. Recruitment followed the same approach as for Stage 3. Participants included: health and social care providers, commissioners and/or end of life care area leads, those in policy-oriented roles, and members of the public. Ahead of both Cafes, participants were sent the draft Guide, alongside information on the topics for discussion on the day, to give them time to read and reflect. On the day, a preliminary presentation of evidence from Stages 1–3 established the context and rationale of the focus for discussion – the draft Guide. Discussion then focused on the Guide and its potential use in practice. Participants were asked to provide feedback on: intended audience, intended use, content, format, and additional issues. To maximise opportunities for input, discussion was conducted in facilitated break-out rooms and participants could also provide asynchronous feedback via email for up to a week after the Cafes. Group facilitators took notes and reviewed the transcript to identify feedback relating to all aspects of the Guide and comments on potential use, as well as limitations. Overall, the response to the Guide was extremely positive; participants were able to readily discern its relevance to practice. The following sections provide detail on feedback received.

### Audience and intended use

We asked if and how the Guide should be targeted for a particular audience. Although participants prioritised a need for commissioners to use the Guide, specific targeting was opposed on the grounds that a necessary focus on systems-level issues would render it less relevant for other audiences. Nor did participants consider the Guide to be useful as a service user-facing document, as this would require content to be very service specific. Rather, the consensus was for the Guide to have general relevance and utility for those working within health and social care settings, relevant across education, reflection and action around the Framework.

### Content and format

Participants recommended explanatory content to include descriptions of terms and concepts, examples, and explicit direction in the form of prompts for consideration. A concentration on clinical activity was eschewed, in favour of guidance regarding intersectional and inclusive approaches. Overall, participants endorsed the Guide’s focus on the Foundations, with the ‘what, ask, action’ format considered a useful structure for facilitating appreciation of the possibilities for relevant work. They found the Guide’s overall style and language both encouraging and enabling. Some participants noted a helpful alignment between the focus on Foundations and the guidance given to ICBs. Two further suggestions were made: that the Guide be made available in both digital and print format and that it enables user input, so that examples could be edited/added to reflect local level priorities.

### Additional topics

As outlined above, during development of the Guide, we added two elements – collaboration and partnership working, and sharing learning. Some participants viewed these as implicitly underpinning the eight Foundations and questioned the need to include them as separate elements. Others found them useful, as reminders of their value in supporting relevant action and how they might be fostered. Participants were divided about our labelling of these elements as ‘foundations’; although considered a fundamental basis of (or ‘foundational’ to) action, on balance, a distinction between these elements and the ‘Foundations’ as set out in the Framework was preferred.

### Recommendations for further engagement with the Framework

Participants suggested other ways to further engagement with the Framework. Some stressed the value of using the Guide in teaching workshops and discussions, especially to help people understand the Framework and potential mechanisms for realising the Ambitions. Others felt that awareness raising of the Framework was required using, for example, webinars, case reports, and an app. Although the current availability of relevant FutureNHS resources was noted, as a password-protected space, participants highlighted their relative obscurity and exclusivity.

**Fig. 2:**
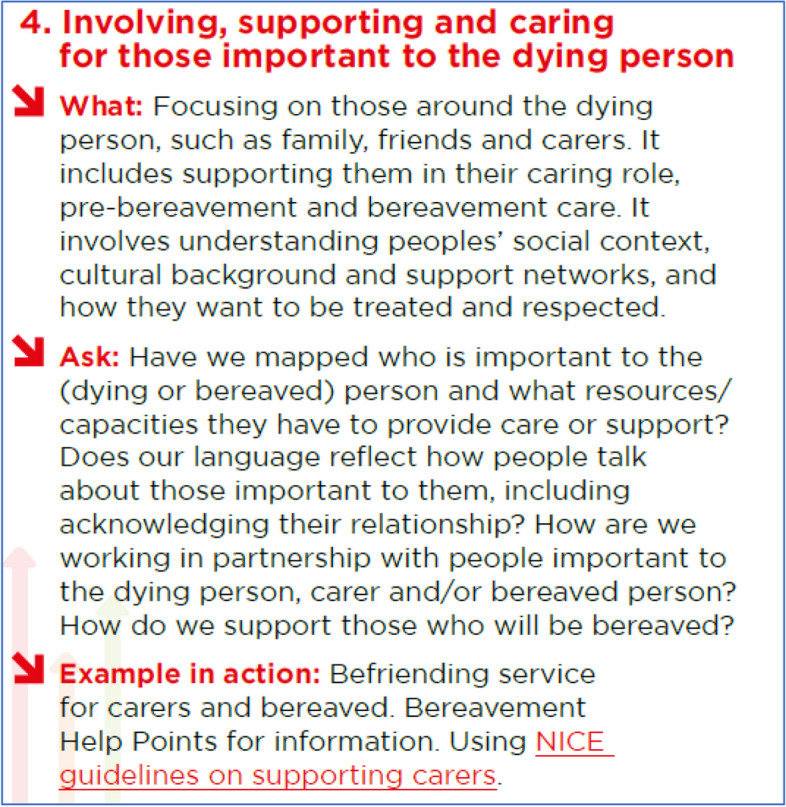
Grab and Go Guide exemplar

Based on the feedback from the Evidence Cafes, we made several key decisions concerning the Guide. Firstly, we kept the intended audience wide, to enable its use by different audiences and in a range of settings. Whilst this may limit the Guide’s impact on specific user groups, core relevance is maintained and the Guide can be used according to different roles and responsibilities. Secondly, we retained content on collaboration and partnership working, and sharing learning, but removed any reference to them as ‘foundations’. Thirdly, a graphic designer worked with us to ensure the guide could be both printable and digital, with embedded hyperlinks. The Guide has a version with copyright license that enables re-use and adaptations with attribution; in this version the prompts and examples are not pre-populated, allowing users to adapt it for their purpose. The final Grab and Go Guide – “Small Steps, Big Vision can be accessed on Open Research Online (ORO) https://oro.open.ac.uk/88013/. Here we provide an example of its content (Fig. 2).

## Discussion

The past thirty years have seen increasing prioritisation of policy as a key component of PEOLC development [32–36], even where national policy exists [37, 38], progress in PEOLC provision has been shown to be limited [39–41], calling into question the role of policy in driving development [37, 42]. A key issue is the extent to which policy is implemented as intended, with a small body of evidence demonstrating the complexities and challenges involved; Whitelaw et al. critically discuss a range of issues that challenge the role of policy in palliative care development [42]. May et al. [43] show how limited resources and competing priorities within both the PEOLC care sector and wider health care system hindered the delivery of Irish national policy. Barker et al. [44] identify challenges to the implementation of United Kingdom (UK) care policy operating at the micro (e.g. patient preferences and priorities), meso (e.g. inadequate resource allocation) and macro (e.g. lack of integrated system working) levels.

The findings of our work add to the limited evidence-base concerning the relationship between policy and PEOLC development. They align with this evidence, suggesting that a linear process from the setting of policy to outcomes cannot be assumed [37]. We show that, in addition to the complexity of the context into which the policy is introduced, implementation can be stymied because of the policy itself. In the case of the Framework, although the Ambitions are endorsed as valid statements of ‘ideal’ PEOLC, their high-level character problematises implementation. An agreed ‘vision’ is set out, but action that might be taken to realise that vision is not. As others have noted, “the likelihood of implementation where policy simply rests on normative ideals is questionable” ([39]; p.89).

In our case, the crux therefore rested on two requirements: (a) proving a roadmap that moves the Ambitions, as normative ideals, to action that might deliver on these ideals. and (b) doing so in ways that fit with the realities of health care provision and working. Our Guide meets these two requirements. In terms of a ‘roadmap’, it unpacks the Foundations, breaking them down both conceptually and practically, therefore helping users to identify relevant action to be taken. Since human knowledge is context-specific and often tacit in nature [45], the ‘ask’ prompt encourages users to consider their own settings and priorities of care provision, thereby facilitating access to this tacit knowledge and understanding. The value of doing so was confirmed throughout our data collection; participants had difficulties realising how work already undertaken or being progressed connected with the Framework.

In terms of accommodating the ‘realities’ of care provision, we needed to strike a careful balance. PEOLC has been described as a ‘wicked’ policy area [46], characterised by complex interdependencies and beset with changing, even contradictory, requirements. Throughout the multiple stages of our data collection, participants communicated a strong sense of sometimes feeling overwhelmed by this complexity. However, the Guide can and does not seek to ‘solve’ all of this complexity; indeed as Lindqvist et al. [32] point out, discrete solutions are not readily available. Instead, it focuses on local-level potential to take small steps, with relatively simple, action-oriented messages. The value of ‘action oriented’ messaging has been demonstrated elsewhere in the policy research field [47]. As a high-order document, the Framework lacks such explicit messaging, something that was repeatedly highlighted as a deficit by our participants. While it does provide some direction in the form of the Foundations as expressions of areas of relevant activity, it cannot speak directly to the multiple, diverse contexts of such activity. It is here that the Guide ‘steps in’ in the form of explanations (the ‘what’) and prompts (the ‘ask’ and ‘examples in action’). We deliberately framed the examples of action as *experience*, as distinct from research findings, reflecting the preference for experience ‘over’ research that has been identified within the knowledge transfer and exchange literature [48]. Moreover, we did not include detailed case studies as examples of possible action, in recognition of the difficulties that can be experienced in the transfer of knowledge across different organisational and practice settings [45].

### Strengths and limitations

One of the strengths of this study is the process through which the object (Guide) was developed, foregrounding an understanding of implementation in practice before creating an object to support implementation and then obtaining feedback on the object. A limitation of this approach, however, is that it relies on the perspectives of those who participate. Since participants were self-selecting, we do not know what would support those who did not attend or express an interest in the Ambitions Framework.

Evidence Cafes are an established method of knowledge exchange with embedded data collection, typically involving a specific item or object, around which discussion coalesces [31]. Developing the Guide as the discussion object enabled us to share research findings – such as the usefulness of focusing on Foundations, case examples, and the themes of sharing learning and collaboration – *and* to gain feedback on the object. Whilst such events tend to be held face-to-face, the online format with facilitated breakout rooms worked well, since we kept participant numbers low and pre-shared the object via email. Participants were also able to provide feedback via email after the event, which enabled flexibility in response and prolonged engagement. A limitation of the method is that its success depends on who attends; the number of commissioners, as well as professionals working in social care, was low. Finally, we are unable to comment on the extent to which the Guide is being used; positive feedback does not necessarily translate into uptake in practice.

## Conclusion

Our extended programme of work helps redress a lack of evidence explicitly connecting policy instruments to practice outcomes [39]. It has produced valuable insight into how policy is made sense of and has contributed towards the implementation of policy (the Ambitions Framework) that has been consistently upheld as a positive instrument for service development [4–8]. The use of Evidence Cafes enabled the creation of a tangible object, already rooted in participant understanding and experience, that could be further refined on the same basis. This was vital for creating a resource that is more likely to be used by the intended audience(s).

### Supplementary Information

Supplementary Material 1.

Supplementary Material 2.

Supplementary Material 3.

Supplementary Material 4.

## Data Availability

No datasets were generated or analysed during the current study.

## Footnotes

1 A summary of our Ambitions Framework research and associated outputs is available at https://wels.open.ac.uk/research/projects/open-thanatology/projects/ambitions-palliative-and-end-life-care.
