# Supplementary material for: Small Steps, Big Vision: using multi-stage qualitative research to develop a grab-and-go guide to support utilisation of the Ambitions for Palliative and End of Life Care framework
Source: BMC Palliat Care. 2024 Jun 14;23:151. doi: 10.1186/s12904-024-01466-8 (PMC11179334; doi:10.1186/s12904-024-01466-8)
Supplement: Supplementary file 1 — Supplementary Material 1. [file 12904_2024_1466_MOESM1_ESM.pdf]

SMALL STEPS, BIG VISION

This is your Grab-and- Go Guide for the Ambitions for Palliative and End of Life Care: A national framework for local action 2021-2026

The framework provides a vision and guidance around six ambitions and eight foundations to improve care. Launched in 2015, it is supported by over 30 partners and embedded in the statuary guidance for ICBs linked to the Health and Social Care Act 2022. Based on in-depth research conducted at The Open University, this guide focuses on the foundations necessary for realising the vision.

For each item, this guide provides: a description, prompts to get you thinking, and examples in action. You can use this guide for your own reflection, to facilitate team discussions, or in work with partners and across systems. We have added two areas to focus on (9&10) to promote collaborative and cross-sector working. This guide helps you to identify areas you can act on - a starting block to turn small steps into the big vision.

1. Personalised care planning

- **What:** Conversations between the person, those close to them and professionals that explore and document the management of their health and care, considering the context of their life, identity and background, as well as family situation. It can include advance care planning and recording of preferences.
- **Ask:** What types of care planning are we already doing? What might improve the quality of the conversations? How could we involve more people in care planning? How equitable and effective is care planning across our system?
- **Example in action:** Advance Care Planning (ACP) education programme across entire locality. Adaptations to ACP documents for people with learning disabilities and culturally sensitive language translations. Universal Principles for Advance Care Planning.

2. Shared records

- **What:** The person and those who are caring for them can access records, such as care plans. It can include electronic systems to facilitate sharing across health and social care providers. It can refer to records created and shared by the person and those important to them.
- **Ask:** What information needs to be shared? How do we currently share information? What barriers do we currently have in accessing information within or across organisations? Does the person, or those important to them, have access to the records in a version that they understand?
- **Example in action:** Treatment escalation plans accessible across different services in an area. Linked up IT systems between acute and primary care using Palliative and End of Life Care Information Standard.

3. Evidence and information

- **What:** The collection of data that can be used locally and nationally to understand service provision and evaluate interventions. It can include using and being involved in research.
- **Ask:** What routine data is being collected and how do we use it? Where are we currently using evidence to inform our actions? Are we collating and listening to a wide range of voices through feedback? Do I know how to make sense of the information I collect?
- **Example in action:** National Audit of Care at End of Life (NACEL) and local mortality data to inform action plan, including address inequities in access. The Integrated Palliative Care Outcome Scale (IPOS) for patient care.

4. Involving, supporting and caring for those important to the dying person

- **What:** Focusing on those around the dying person, such as family, friends and carers. It includes supporting them in their caring role, pre-bereavement and bereavement care. It involves understanding peoples' social context, cultural background and support networks, and how they want to be treated and respected.
- **Ask:** Have we mapped who is important to the (dying or bereaved) person and what resources/ capacities they have to provide care or support? Does our language reflect how people talk about those important to them, including acknowledging their relationship? How are we working in partnership with people important to the dying person, carer and/or bereaved person? How do we support those who will be bereaved?
- **Example in action:** Befriending service for carers and bereaved. Bereavement Help Points for information. Using NICE guidelines on supporting carers.

5. Education and training

- **What:** Education of staff and/or volunteers, ensuring they are competent in their knowledge about good end of life care and that this is maintained. It includes focusing on skills sets and quality assurance of training.
- **Ask:** What do people already know about end of life care? Are we making good use of free educational resources? How can we invest in and embed training across our system?
- **Example in action:** National e-learning programme End of Life Care for All (e-ELCA) with over 120 free online modules. End of Life Care Facilitator providing education across acute and community providers in an area, recording of who is trained. Palliative Care team using Ambitions Framework to structure education for all Trust staff.

6. 24/7 access

What: System-wide access to end of life care support, including symptom management, at all hours and days of the week. This does not mean that each service must always be available, but that there is accessible and equitable coverage across a system/area to meet needs.

Ask: Where would we direct someone out-of-hours? Who might need access to services out-of-hours and why? Are existing out-of-hour services accessible for all groups of people?

Example in action: Hospice 24/7 helpline accessible to staff, individuals, and families. Use of Ambitions self-assessment to identify gaps in service provision.

7. Co-design

What: Designing services in collaboration with people who have personal and professional experience of palliative and end of life care, including patients and carers. Methods of engagement can be creative, participatory, and experience-based. Co-design should embrace diverse views and not be limited to service provision commissioning decisions.

Ask: What knowledge do I have about co-design? Who are we involving (individuals and organisations)? How do people want to be engaged with? Who is best placed to facilitate co-design processes? In what ways do we actively involve people in designing a service and making decisions about how we provide care? How are we learning from people's lived experiences, their support needs, and what care and support they access and want to access?

Example in action: Project focusing on bereaved carers providing Trusts with insights on how to improve physical and support environments for end of life care and post-death experiences.

8. Leadership

What: People and organisations who have the capacity and remit to identify what needs to happen, communicate visions, and motivate others to realise the Ambitions. Leadership is not limited to those with formal positions of authority.

Ask: Who am I expecting to take this forward? In what ways can I instigate change? How can I support others to realise the Ambitions? What strategic leadership is needed for system-level changes?

Example in action: Trusts showcase their ongoing development of services when writing local reports and presentations, highlighting how using the Framework has helped them identify gaps and goals. Team Leaders include the framework in regular meetings, fostering working cultures that support the Ambitions. Development of compassionate leadership that supports staff wellbeing and resilience.

9. Collaboration and partnership working

What: Working with others (within and beyond your organisation) on a common goal. Partnership may involve sharing resources and responsibilities.

Ask: Who do we need to be working with? How knowledgeable are our potential partners with the Ambitions or end of life care? Have we discussed our expectations about roles and responsibilities? What barriers do we have to working together?

Example in action: Hospice uses the Framework when working with partners to facilitate sharing goals and best practice under different themes, with dedicated time for networking and relationship building.

10. Sharing learning

What: The process of exchanging information and insights with others with the intention of achieving a common objective or goal over time. It can involve acquiring new knowledge and skills as well as becoming part of a community.

Ask: Where can I find out about what others are doing? How can I tell others about what my organisation does and lessons learned? In what ways do I reflect on what others have done and try it out in my own practice? Am I open to learning from people with different experiences, perspectives, and roles (including from non-professionals)? What is needed to facilitate more intentional learning in the environments I am part of?

Example in action: Involvement in workshops and networking events, including sharing documents and case studies (e.g. FutureNHS). Feedback learning to one's own team through meetings and learning events.

Interested in knowing more? Link up with your local ICB or palliative care team. Your local contact is:

Footnote: This guide has been developed by Dr Erica Borgstrom, Claire Henry MBE, Dr Joanne Jordan and Dr Una St-Ledger of Open Thanatology at The Open University. Marie Curie funded this research. For more information project findings and outputs, visit: https://tinyurl.com/ambitionsframework.
For more resources about the Ambitions, check FutureNHS.
This guide does not replace statutory or NICE guidelines

Version 1 - March 1 2023 - Creative commons CC-BY

The Open University
OPEN THANATOLOGY
FUNDED BY Marie Curie Care and support through terminal illness
