# Supplementary material for: Small Steps, Big Vision: using multi-stage qualitative research to develop a grab-and-go guide to support utilisation of the Ambitions for Palliative and End of Life Care framework
Source: BMC Palliat Care. 2024 Jun 14;23:151. doi: 10.1186/s12904-024-01466-8 (PMC11179334; doi:10.1186/s12904-024-01466-8)
Supplement: Supplementary file 2 — Supplementary Material 2. [file 12904_2024_1466_MOESM2_ESM.docx]

**Project title:** **Examining the Ambitions Framework: in-depth case study analysis and future directions**

**PI: Prof Erica Borgstrom, The Open University**

**Project Funder: Marie Curie (grant MCSGS-21-602)**

**Evidence Café – Prompt Questions for Facilitators in Breakout Rooms**

Purpose of the breakout room discussion is to get participants to focus on the ‘Grab and Go’ guide. These are suggested prompts to get the discussion flowing:

Use:

- Can you envision yourself using this? If so, how? If not, why not? (Enquire a bit about their context and role to help make sense of their answer) When might you use it?
- Who else do you think might find this useful?

Content:

- Is the content clear to you?
- Anything missing?
- What kinds of prompts do you find helpful?
- This guide focuses on the foundations within the Ambitions framework – how familiar are you with them? How useful do you find them?

Format:

- What size would be helpful?
- What medium would be most useful? Online, printed? Website?
- How long would you ideally like this guide to be?
- What other information would you need to help you make use of this?

General:

- What would make it better?
- What do you struggle with when implementing the Ambitions?
- What do you find easy when it comes to implementing the Ambitions?
- Whose responsibly do you think it is to realise the Ambitions? How might they be better served to do this?
- What other kinds of events or information would you find useful? Where/how would you want to access them?
