# Supplementary material for: Small Steps, Big Vision: using multi-stage qualitative research to develop a grab-and-go guide to support utilisation of the Ambitions for Palliative and End of Life Care framework
Source: BMC Palliat Care. 2024 Jun 14;23:151. doi: 10.1186/s12904-024-01466-8 (PMC11179334; doi:10.1186/s12904-024-01466-8)
Supplement: Supplementary file 3 — Supplementary Material 3. [file 12904_2024_1466_MOESM3_ESM.docx]

**Project title:** **Examining the Ambitions Framework: in-depth case study analysis and future directions**

**PI: Prof Erica Borgstrom, The Open University**

**Project Funder: Marie Curie (grant MCSGS-21-602)**

**Focus Group Guide**

Introduction: we want to ensure we capture everyone’s thoughts so please can we ensure the following is agreed

- important that everyone has an opportunity to contribute; if you would like to say anything while others are talking, please feel free to raise your hand (using the Teams ‘button’ and/or write comments in the chat box) and lower your hand after speaking. If possible, keep mic muted when not speaking.
- We are aware that everyone will have different levels of knowledge about and use of the Ambitions Framework. The focus group is not about ‘testing’ your knowledge – instead, we want to know more about what think about it and if and how they use it. There are no ‘right or wrong’ answers, all are valid.
- feel free to agree and disagree with one another, but always respectfully
- confidentiality – conversation not to be discussed outside the focus group
- audio-recorded – to ensure accurate record of conversation; transcription made of each focus group conversation, which is fully anonymised; all welcome to a copy of their own contribution to the focus group (anonymised)
- check that all participants have signed the Consent Form. Check verbal consent and remind them that they can leave the focus group at any time.
- initial round of BRIEF introductions – who the participants are, where they are located, what is their role. Chair and co-facilitator model this for the group.

Question 1: We are now showing a slide with all six Ambitions listed. Take a moment to read them. We will ask you a few questions about these ambitions. No response is too silly here – part of helping people use documents like this is to ensure people can understand them.

- What does each one mean to you? (Is it clear to you what they each mean? Do they make sense to you?)
- Do you think there is an implicit order to these or hierarchy? I.e. does one need to happen before the other can happen?
- Is anything missing? How do you think they relate to each other?
- Which of these Ambitions do you think are most pertinent to your role/work? Why?

Question 2: We’d like to learn more about your understanding of two of the Ambitions in particular - Ambition 1 and Ambition 6. We are focusing on these two Ambitions because the findings of our previous survey showed these to be the most and least used of the Ambitions.

*Ambition 1: Each person is seen as an individual*.

- What does this Ambition mean to you? (if not covered in Q1)
- who do you think this Ambition is targeted at?
- how do you think it is meant to be/can be used?
- how relevant is it to your own role/work and why/why not?

*Ambition 6: Each community is prepared to help.*

- What does this Ambition mean to you? (if not covered in Q1)
- What do you think ‘community’ refers to in this ambition?
- who do you think this Ambition is targeted at?
- how do you think it is meant to be/can be used?
- how relevant is it to your own role/work and why/why not?

Question 3: Have you used the Framework in any of your work? *Probe in relation to the particular role/work of the different groups of participants.*

**If so**:

- Which Ambitions did you use and why?
- Overall experience of this use - positive / negative and why? *Probe for: how far and in what ways did use of the Ambition(s) enable/help in your work; what challenges did use of the Ambition(s) pose?*

***If not***, why not? *Probe for: didn’t know about it; not thought it useful/helpful to use.*

Question 4: How do you think the Ambitions Framework could be further developed in relevance to your future work?

- adding to/changing the content e.g. new/different Ambitions? the number/range of Ambitions
- the differential value/relevance of the six Ambitions?
- Would it make a difference if the Framework formal policy? Why/why not?
- Any additional resources to undertake the work?

Question 5: Prior to this focus group, how familiar did you feel with the Ambitions Framework? Has this changed during the discussion, and if so, how?

Question 6: Are they any other issues that you see as relevant to our discussion that we have, so far, not discussed?

Thanks and close down.
