# Supplementary material for: Small Steps, Big Vision: using multi-stage qualitative research to develop a grab-and-go guide to support utilisation of the Ambitions for Palliative and End of Life Care framework
Source: BMC Palliat Care. 2024 Jun 14;23:151. doi: 10.1186/s12904-024-01466-8 (PMC11179334; doi:10.1186/s12904-024-01466-8)
Supplement: Supplementary file 4 — Supplementary Material 4. [file 12904_2024_1466_MOESM4_ESM.docx]

**Project title:** **Examining the Ambitions Framework: in-depth case study analysis and future directions**

**PI: Prof Erica Borgstrom, The Open University**

**Project Funder: Marie Curie (grant MCSGS-21-602)**

**Case Study Interview Guide (Stage 1 of Project)**

Introduction: issues to cover

- Remind no ‘right or wrong’ answers
- Request to audio-recorded – to ensure accurate record of conversation; transcription made of each interview (if on MS Teams this will be autogenerated initially), which is later fully anonymised; welcome to have a copy of their transcript (if on MS Teams they may be able to access the automated version after the session)
- check they have signed the consent form and consent to go forward with the interview

Question 1: How familiar are you with the Ambitions Framework (e.g. document)? How did you learn about it? [Note: if not a survey respondent, you can start this question asking if they are familiar with AF]

*Prompt: check if the Framework has been used in relation to one or more pieces of work; if used more than once, ask all questions in relation to each instance. Clarify their role in these (e.g. initiate activity, coordinate, etc).*

Question 2: What were your reasons for using the Framework - the initial thinking of why it would be useful / helpful? What influenced your use of the framework? *Prompt for: which Ambitions were used; why these Ambitions?*

Each person is seen as an individual (Ambition 1)

Each person gets fair access to care (Ambition 2)

Maximising comfort and wellbeing (Ambition 3)

Care is coordinated (Ambition 4)

All staff are prepared to care (Ambition 5)

Each community is prepared to help (Ambition 6)

Opening question 3: Summary description of case study service/example to get an overall sense of what was done and to understand the basic context of remainder of the discussion. *Prompt for:*

- *New service / enhancement of previously existing service etc.?*
- *Focus of the service – geographical area, setting and population?*
- *Aims of the service?*
- *Funding of the service?*
- *Organisations / stakeholders involved? Why these stakeholders (and not other potential ones); how / when were each involved in the work - their respective roles and responsibilities? How were patients/service users/clients and/or the public involved?*
- *Use of other policy, guidance, or frameworks when designing or delivering the service?*
- *Stage of the work: ongoing / completed / now embedded in standard provision?*

Question 4: How has the Framework been used? *Prompt for: prospectively/ (i.e. after publication in 2015) / retrospectively; in what way(s) (e.g. guiding principles (values) / service design / business case / commissioning / quality improvement / audit etc.).*

Question 5: Has the service been reviewed or evaluated? **If yes**, in what way (e.g. audit, feedback surveys, formal evaluation etc.) **If yes**, what were the findings, and can this be shared with us (e.g. send a report)? **If not reviewed**, reasons? **If not reviewed**, have you had any other feedback / information that gives you an idea of how well the work has gone?

Question 6: How, if at all, did use of the Framework help in your work? What did its use enable/allow that might otherwise have been difficult / not possible? Why do you think it helped? *Prompt for: did it help show what could be aspired to; provide a common aim / direction; provide an explicit way of working (together)?*

Question 7: How, if at all, did use of the Framework inhibit or create any problems / challenges for you in your work? *Prompt for: how successful was the translation of the aspiration (ambition) into practice; was the aspiration (ambition) understood / applied by all involved in the way you envisaged; did the Ambition end up being too ambitious / nebulous?*

Question 8: Aside from the content of the Framework itself, what other factors / issues are you aware of that impacted on your use of the Framework? *Prompt for e.g.: available resources to support any work; opportunities for meaningful partnership working; pressure of ongoing workload (including re: impact of Covid)*.

Question 9: How do you think the Framework could be developed to enhance its role and relevance to your work? *Prompt for e.g.: making the Framework formal policy; the number / range of Ambitions; the differential value / relevance of the six Ambitions; additional resources to undertake the work*.

Question 10: Based on your experiences, what are the next steps for you? Would you be inclined to use the Framework in future work?

**If so**, in what way(s)? *Prompt for: reason(s) for use of the Framework in the way(s) described*. If not, why not.

Question 11: Are they any other issues that you see as relevant to our discussion that we have, so far, not talked about?

Thanks and close down.
